# The Impact of Gallic Acid Binding on the Foam and Interfacial Properties of Whey Protein Isolate Under Weak Acidic Conditions

**DOI:** 10.3390/foods14183209

**Published:** 2025-09-15

**Authors:** Fuchao Zhan, Jing Li, Bin Li

**Affiliations:** 1 College of Food Science and Technology, Nanjing Agricultural University, Nanjing 210095, China; 2 College of Food Science and Technology, Huazhong Agricultural University, Wuhan 430070, China; lijingfood@mail.hzau.edu.cn; 3 Shenzhen Institute of Nutrition and Health, Huazhong Agricultural University, Shenzhen 518000, China; 4 Shenzhen Branch, Guangdong Laboratory for Lingnan Modern Agriculture, Genome Analysis Laboratory of the Ministry of Agriculture, Agricultural Genomics Institute at Shenzhen, Chinese Academy of Agricultural Sciences, Shenzhen 518000, China

**Keywords:** whey protein isolate, gallic acid, complexes, structure, interfacial properties, foam properties

## Abstract

The interfacial and foam properties of proteins can be enhanced by altering the interactions between polyphenols and proteins. The aim of this study was to determine the influence of gallic acid (GA) on the structural properties of whey protein isolate (WPI), specifically focusing on particle size, potential, and surface hydrophobicity, as well as the subsequent alterations in its interfacial and foam properties when utilized as a foaming agent. An increase in turbidity and a decrease in particle size suggested the formation of a soluble complex between GA and WPI at a pH of 6. The results from fluorescence spectroscopy and surface hydrophobicity analyses indicated that the primary interactions between GA and WPI are characterized by hydrogen bonding and hydrophobic interactions. The reduction in particle size enhances the capacity of WPI/GA complexes to lower the surface pressure, thereby demonstrating significant efficacy at the macroscopic scale. Furthermore, the structural connectivity of GA facilitates the formation of a stable interfacial film at the air–water interface by WPI/GA, resulting in high foam stability at a macroscopic level. This research contributes to a deeper understanding of the application of protein–polyphenol complexes as surfactants and provides theoretical support for their use in food applications.

## 1. Introduction

Foam, a colloidal dispersion composed of bubbles within a continuous liquid phase, plays a crucial role in enhancing the structure of various food products, including baked goods, confections, and ingredient formulations [1,2]. Whey protein isolate, a by-product commonly derived from cheese or casein production, is frequently employed to boost the protein content in these foods while also improving their foamability and foam stability [3,4]. The formation of foam is significantly influenced by the adsorption of protein-based surfactants, which enhance the reduction in surface tension; facilitate the generation and maintenance of large interfacial areas; and increase interfacial viscosity or elasticity. Additionally, variations in protein structure critically impact their capacity to lower surface tension and create viscoelastic films. Consequently, the protein structure of whey protein isolate is pivotal in determining its foam properties. In recent years, food scientists have sought to improve the interfacial and foam characteristics of proteins by modifying their structure through various approaches. These include physical treatments (such as heat, high pressure, or ultrasound), chemical alterations (like pH shifts or covalent cross-linking), and enzymatic modifications, all aimed at broadening their applicability for foam-related applications [5,6].

It is widely recognized that polyphenolic bioactive compounds can interact with and bind to proteins, effectively modulating protein structure and function while enhancing the stability of associated bioactive factors, which has gradually become a hotspot in food academic research [7,8]. Interactions between proteins and polyphenols primarily occur through covalent and non-covalent bonding, which can influence proteins’ structure and subsequently improve their interfacial or foaming properties. For instance, Pei et al. developed an anionic phytate–whey protein complex, demonstrating that this complex exhibited a superior reduction in interfacial tension and enhanced electrostatic bridging compared to those for whey protein alone [9]. Similarly, Pan et al. formed covalent complexes between chlorogenic acid and proteins under alkaline conditions, leading to increased interfacial adsorption capacity and a denser interfacial layer [10]. In addition to the enhancements provided by covalently bonded protein–polyphenol complexes, non-covalently bonded protein–phenol complexes have also been shown to significantly improve the interfacial properties of proteins. For example, Han et al. found that epigallocatechin gallate (EGCG) can enhance the foaming properties of whey protein isolate (WPI) through non-covalent interactions [11]. Additionally, Li et al. reported that proanthocyanidins bind to lactoferrin through hydrogen bonding and hydrophobic interactions, resulting in improved foam stability [12]. Overall, it is evident that binding with polyphenols serves as a straightforward and rapid method for modifying protein structure. Therefore, gaining a deeper understanding of protein–polyphenol interactions and their effects on the functional properties of proteins is essential for expanding their applications within the food industry.

In this study, a multispectral analysis was employed to investigate the interaction between gallic acid (GA) and whey protein isolate (WPI). Gallic acid (GA) was selected based on its excellent water solubility and antioxidant capacity [13]. Furthermore, as a small-molecule polyphenol with a limited number of binding sites, GA is less likely to induce excessive protein aggregation or cross-linking, thereby avoiding functional deterioration. We examined how GA binding affected the structural properties of WPI, specifically focusing on parameters such as particle size, zeta potential, and surface hydrophobicity. Additionally, the relationship between the interfacial properties of the WPI/GA complexes and the macroscopic foam properties was explored. The findings from this research are anticipated to provide valuable insights into the connections between polyphenol–protein interactions, interfacial properties, and foam characteristics. This knowledge could facilitate the development of new functional food ingredients and enhance the applications of protein–polyphenol complexes in foam-based food products.

## 2. Materials and Methods

### 2.1. Materials

Whey protein isolate (WPI) with a solubility > 50 mg/mL was purchased from Sigma Chemical Co. (St. Louis, MO, USA). Gallic acid (GA, MW 170.12 g/mol, 97.5%) was purchased from Shanghai Aladdin Biochemical Technology Co., Ltd. (Shanghai, China). NaH_2_PO_4_·2H_2_O and Na_2_HPO_4_·12H_2_O were purchased from Sinopharm Chemical Reagent Co. (Shanghai, China).

### 2.2. Preparation of the Whey Protein Isolate/Gallic Acid Complexes

The complexes were prepared based on our previous studies [14], with modifications. Phosphate-buffered solution (PBS, pH 6, 10 mmol/L) was prepared using NaH_2_PO_4_·2H_2_O and Na_2_HPO_4_·12H_2_O. WPI was dissolved in PBS (pH 6, 10 mmol/L) using a magnetic stirrer (JJ-1, Shanghai, China) at room temperature (RT, 27–29 °C) for 2 h at a concentration of 5% (*w*/*v*), followed by fine-tuning of the pH to 6 using 0.1 mmol/L HCl. Sodium azide (NaN_3_) was added to prevent microbial growth. The WPI solution was placed in the refrigerator (4 °C) overnight for the next step. GA solution (2%, *w*/*v*) was prepared by dissolving GA power in PBS (pH 6, 10 mmol/L) under continuous stirring at room temperature (RT, 27–29 °C) for 1 h. The WPI/GA complexes were prepared by mixing WPI solution and GA solution with continuous stirring at room temperature (RT, 27–29 °C). The final concentration of WPI was 1% (*w*/*v*), and that of GA was 0, 0.1, 0.3, and 1% (*w*/*v*), respectively. Then, the WPI/GA complex solutions were stored overnight at 4 °C.

### 2.3. Determination of Particle Size and Zeta Potential

The particle size and zeta potential of the prepared samples were analyzed using a Nano ZS Nanoparticle Size Potential Analyzer (Zetasizer Nano ZS90, Malvern, UK). The WPI/GA complex solutions (mass ratios of 1:0.1, 1:0.3, 1:0.5, and 1:1, respectively) were diluted 10-fold using PBS (10 mM, pH 6.0, final WPI concentration of 0.1%, *w*/*v*) prior to testing. The refractive indexes of the WPI and PBS buffer solutions were 1.46 and 1.33, respectively. All samples were determined with three replicates.

### 2.4. Determination of Turbidity

The turbidity of the WPI/GA complexes was assessed using a UV-Vis Spectrophotometer, as described in our previous study [15]. The interaction between GA and WPI affects the transmission of UV light in the mixed solution. A total of 200 μL of the WPI/GA complexes with different GA concentrations was transferred into 96-well plates, and the absorbance of each sample was measured at 600 nm using a full-wavelength enzyme labeler (Multiskan Sky High, Thermo Scientific, Waltham, MA, USA). The turbidity of the samples was calculated as 100-T%, where T represents the absorbance recorded at 600 nm. A PBS solution was used as the blank for calibration. All measurements were carried out at room temperature (25 °C), and three replicate experiments were performed.

### 2.5. Determination of Surface Hydrophobicity

The surface hydrophobicity of different samples was measured according to the method in our previous studies [16], with some modifications. The WPI/GA complex samples were diluted 200-fold with PBS (10 mM, pH 6.0) to a final WPI concentration of 0.005% (*w*/*v*). ANS solution (40 μL, 8 mmol/L, pH 6.0) was added to 4 mL of the WPI/GA complex solution and mixed in a dark room for 5 min. The fluorescence spectra of the WPI/GA complex were measured using an F-4600 fluorescence spectrometer (Hitachi Corp., Tokyo, Japan) with the excitation wavelength set to 390 nm and the emission wavelength range set from 400 nm to 600 nm. The maximum area of the fluorescence spectra was adjusted to the buffer, and the relative difference in the area represented the hydrophobicity (S_0_) of the sample. Each measurement was performed at room temperature (25 °C) and repeated three times for accuracy.

### 2.6. The Spectrofluorometric Analysis

Fluorescence spectroscopy of WPI and the WPI/GA complexes was measured using a spectrofluorometer (F-4600, Hitachi, Japan). The concentration of whey isolate protein (WPI) was diluted with PBS buffer to 0.5% (*w*/*v*), and the mass ratios of the WPI/GA complexes were 1:0, 1:0.1, 1:0.3, 1:0.5, and 1:1 in a volume of 10 mL. The excitation wavelengths were 280 nm and 290 nm, respectively, and the emission spectra were scanned in the range of 300–500 nm with a scanning speed of 1200 nm/min and a slit width of 10 nm.

### 2.7. Determination of Surface Tension and Dilatational Rheology

#### 2.7.1. Surface Tension

The surface tension (γ) of the WPI/GA complexes was measured using a Tracker-H automated suspension droplet tensiometer (Tracker, Teclis Instruments, Tassin, France) according to the method mentioned in our previous study [17]. The WPI/GA solution (20 mL) was placed in a cuvette for 30 min prior to the experiment. To ensure the accuracy of the measurement, a steel needle was fixed to the syringe, which was secured into the groove of the instrument. A steel needle was then inserted into the rectangular cuvette containing the sample, and the syringe was pressed to form a bubble. High-speed cameras were used to record changes in the bubbles throughout the process The constant volume of the bubble was set to 10 μL, and the measurement was carried out at a constant temperature of 25 °C for a duration of 180 min. The variation in γ with adsorption time (t) was calculated through a numerical analysis of the images according to the Young–Laplace equation using the system software, and then the surface pressure (π) was calculated from the following equation:

(1) $$\pi =\gamma_{0}-\gamma$$

where γ_0_ is the surface tension of the PBS buffer (25 °C, γ_0_ = 72.0 ± 0.5 mN/m), and γ is the surface tension of the sample to be measured.

#### 2.7.2. Interfacial Rheology Measurements

The dilatational rheology of the air–water interface formed by the WPI/GA complex was analyzed using a Tracker-H automated droplet tensiometer. The parameters of the surface viscoelastic modulus (E), surface viscous modulus (E_v_) and surface elastic modulus (E_d_) were evaluated. The frequency was set to 0.1 Hz, and the amplitude was fixed at 10%. A total of five oscillation cycles and five blank cycles were measured throughout the process, and the recording time was 180 min.

### 2.8. Foamability and Stability

The foam properties of the WPI/GA complexes were evaluated according to our previous research [18]. WPI/GA complex solutions with different GA concentrations were poured into a glass cup at room temperature (25 °C). A high-speed disperser (T25, IKA, Staufen, Germany) was placed in the center of the glass cup, and the foam was prepared at 10,000 rpm for 2 min. Freshly prepared foam was quickly transferred into a separate measuring cylinder (50 mL). The foam volume was recorded at 0 min, 2 min, and 60 min.

The foamability (FA) and foam stability (FS) of the samples were calculated as follows:

(2) $$FA=\frac{V_{2}}{20}\;*\;100\%$$

(3) $$FS=\frac{V_{60}}{V_{2}}\;*\;100\%$$

where V_2_ is the foam volume of the sample at 2 min, and V_60_ is the foam volume of the sample at 60 min.

### 2.9. Statistical Analysis

Data from all parallel experiments were analyzed through an ANOVA using SPSS version 21.0 (IBM, New York, NY, USA). Differences between samples and treatment effects were assessed using Duncan’s multiple range test (*p* < 0.05).

## 3. Results and Discussion

### 3.1. Particle Size and Potential Analysis

**Figure 1:**
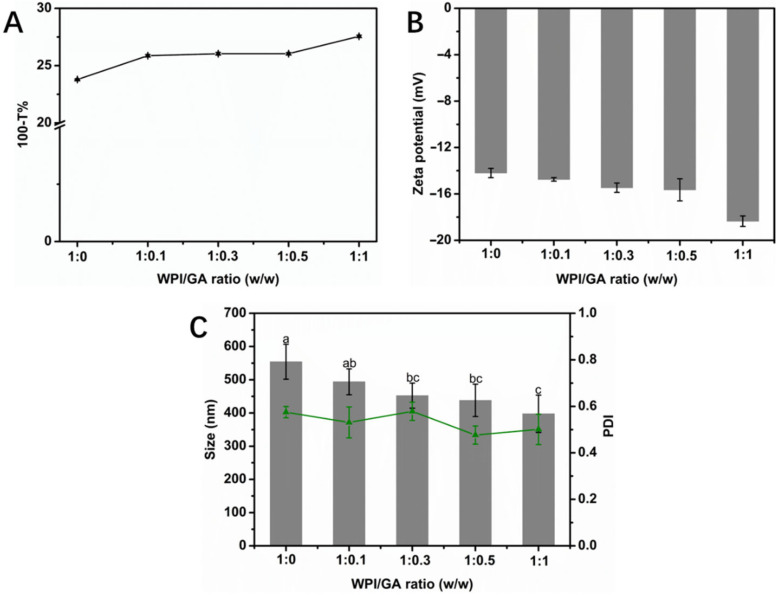
The change in turbidity (**A**), zeta potential (**B**), and particle size and PDI (polydispersity index) (**C**) of WPI/GA complexes with different mass ratios.

To prevent the formation of an unstable colloidal suspension, the WPI/GA complex was prepared by adding GA dropwise to WPI while gently stirring. The turbidity of the WPI/GA complex solutions at various mass ratios was measured, with the WPI concentration fixed at 1% (*w*/*v*). As illustrated in Figure 1A, the turbidity of the WPI/GA complex solutions increased with an increasing GA concentration (ranging from 0% to 1%). Notably, no significant precipitation or sedimentation was observed after one day at room temperature, indicating that the complexes formed between WPI and GA exhibit excellent resistance to gravitational effects. The variation in the particle size and the charge of the WPI/GA complex are depicted in Figure 1C. The average diameter of the WPI/GA complexes decreased from approximately 550 nm to 400 nm as the GA concentration increased, and the complexes displayed a relatively polydisperse distribution (with a polydispersity index of around 0.5). According to previous studies, polyphenols can “bridge” protein molecules, facilitating stable interactions between them [16]. The phenolic hydroxyl group of GA binds to the hydrophobic region (aromatic amino acids) of WPI, weakening the hydrophobic interactions between proteins and reducing nonspecific aggregation [19]. In addition, GA is negatively charged (pKa ≈ 4.5) and produces electrostatic repulsion from WPI at a pH = 6.0, inhibiting particle aggregation further [20]. Consequently, the average particle size of the WPI/GA complexes was significantly smaller than that of the individual WPI molecules. Similarly, earlier researchers indicated that the presence of proanthocyanidins reduced the size of complexes formed of pea proteins [21].

As shown in Figure 1B, both single WPI and the WPI/GA complexes had a negative charge (ranging from −14 to −19 mV) at a pH = 6. The negative surface charge of the WPI/GA complexes increased progressively with higher GA concentrations. This trend can be attributed to the fact that WPI is negatively charged at a pH = 6, which is well above its isoelectric point (around a pH = 4.8). Additionally, pure GA solution (without protein) also displays a negative charge at a pH of 6. This observation further supports the notion that the binding of WPI to GA strengthens as the GA concentration rises, resulting in a gradual increase in the overall negative charge on the WPI’s surface.

### 3.2. Protein Surface Hydrophobicity

**Figure 2:**
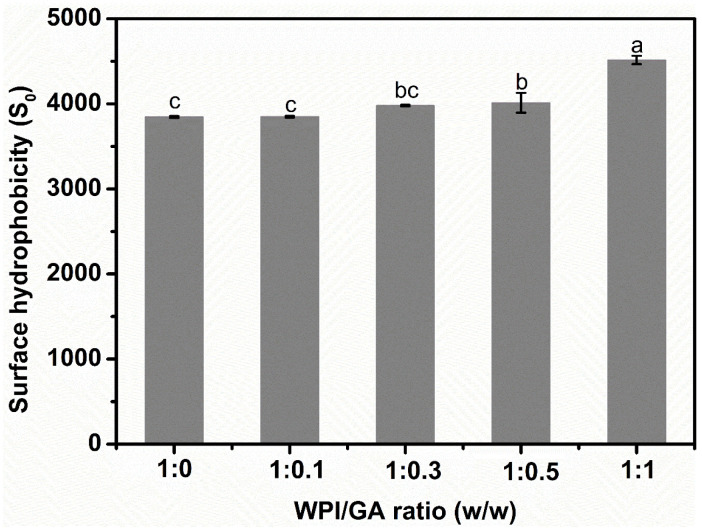
The change in surface hydrophobicity (S_0_) with different mass ratios for WPI/GA complexes.

The surface hydrophobicity of proteins is determined by the number of non-polar amino acid residues exposed to the surrounding aqueous phase, which can be assessed using ANS fluorescent probe methods. As shown in Figure 2, the surface hydrophobicity of WPI progressively increased as the GA concentration rose from 0 to 1%. This trend suggests that GA treatment enhances the hydrophobicity of WPI molecules. One possible explanation is that the interaction between GA and WPI introduces additional hydrophobic groups, leading to an overall increase in the surface hydrophobicity of WPI. Additionally, GA may promote the unfolding of the WPI’s molecular structure, thereby exposing previously hidden hydrophobic groups.

### 3.3. Protein Internal Fluorescence

**Figure 3:**
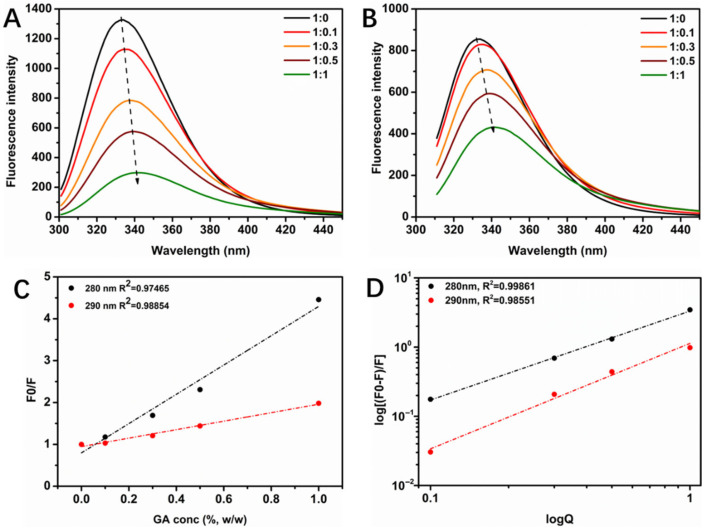
Fluorescence emission spectra of WPI/GA complexes with different mass ratios at excitation wavelengths of (**A**) 280 nm and (**B**) 290 nm. Corresponding Stern–Volmer plots are shown in (**C**); the double logarithmic regression plots (**D**).

Proteins possess inherent fluorescent properties, mainly due to the fluoresce of tryptophan, tyrosine, and phenylalanine residues at specific excitation wavelengths [22]. Tyrosine and tryptophan residues can both be excited and fluoresce at 280 nm, while only tryptophan residues fluoresce at 290 nm [23]. Intrinsic fluorescence emission spectroscopy was employed to investigate the structural changes induced by the interaction between WPI and GA. As shown in Figure 3A,B, the fluorescence intensity of the WPI/GA complex gradually decreased with an increasing GA concentration when the excitation wavelength was 280 nm. Meanwhile, the maximum fluorescence emission wavelength for WPI was 333 nm, which was significantly red-shifted upon binding with GA. Similar results were observed at the 290 nm excitation wavelength, where the fluorescence intensity also decreased and the maximum emission wavelength of WPI showed a significant red shift. The decrease in the intrinsic fluorescence intensity at both excitation wavelengths suggests that both tryptophan and tyrosine residues of WPI are involved in binding to GA, resulting in fluorescence quenching. As seen in Figure 3C,D, the fluorescence quenching of WPI at an excitation wavelength of 280 nm did not overlap with that at an excitation wavelength of 290 nm, further supporting the involvement of both tryptophan and tyrosine residues in the binding with GA.

The *K_q_* values at 280 nm and 290 nm excitation wavelengths are summarized in Table 1. Static quenching occurs if the *K_q_* value exceeds 2 × 10^10^ L/(mol·s) (maximum limiting diffusion quenching constant) at ambient temperature. Calculations based on the equation yielded that all *K_q_* values obtained in this experiment were greater than 2 × 10^10^ L/(mol·s), indicating that the type of quenching between WPI and GA is static quenching due to complex formation. Subsequently, the double logarithmic equations were fitted to (F_0_ − F)/F and Q. As shown in Table 1, the intercept and slope of the curves are log *K_a_* and *n*. At an excitation wavelength of 280 nm, *K_a_* and *n* were 3.32 L/mol and 1.52, respectively, suggesting that the two were combined by weak intermolecular forces, such as hydrogen bonding, and that there was about one binding site available for GA in the fluorescent moiety of each protein.

### 3.4. Measurement of Interfacial Rheological Properties

**Figure 4:**
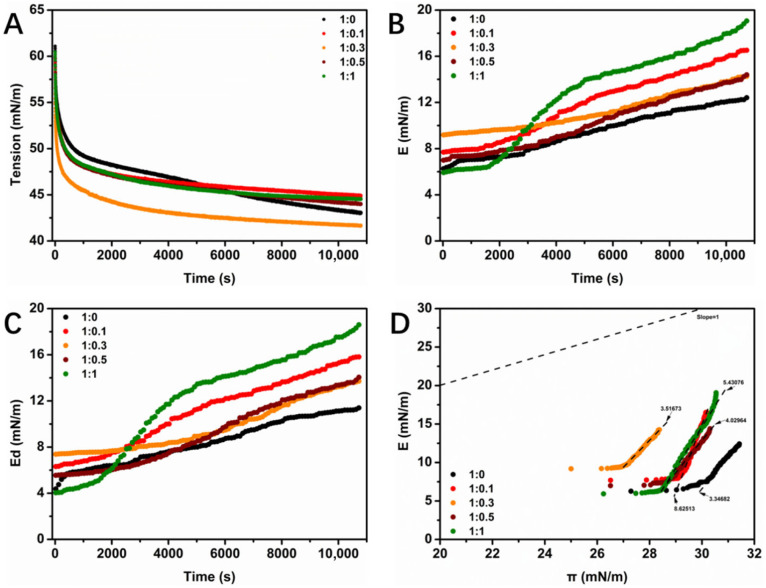
The change in the surface tension (γ) (**A**), surface dilatational modulus (**B**), and elastic modulus (**C**) with time (t) for the WPI/GA complexes; E as a function of π for WPI/GA complexes at the air–water interface (**D**).

Dynamic surface tension measurements were conducted to assess the effect of GA on the adsorption capacity of WPI at the interface. As shown in Figure 4A, the surface tension decreased with an increasing adsorption time, eventually approaching an equilibrium state within 180 min. This reduction in surface tension indicates that the adsorption of WPI/GA complexes at the air–water interface gradually increased until it reached an equilibrium state. The surface tension curves for both WPI and the WPI/GA complexes followed similar trends over time. At equilibrium, the interfacial tension of the WPI/GA complex was slightly higher than that of the single WPI sample when the WPI/GA mass ratio was either less than or greater than 1:0.3. The lowest interfacial tension at equilibrium was observed at a WPI/GA mass ratio of 1:0.3, suggesting that the WPI/GA complex exhibited the strongest ability to reduce the surface tension in this case. This phenomenon suggests that the surface activity of WPI can be increased at a GA concentration of 0.3% (*w*/*v*). This may be due to the fact that GA increases the surface hydrophobicity of the WPI/GA complexes, improving their adsorption properties. Additionally, the presence of GA decreases the particle size of the WPI/GA complexes, and smaller particles tend to have better adsorption properties.

Previous studies have shown that excellent interfacial tension reductions can lead to enhanced foam properties [24]. However, the results from foam performance tests do not always align with surface tension data. For instance, a similar discrepancy was observed in our previous study in the interfacial and foam properties of sodium caseinate and GA complexes [25]. The interactions between proteins and polyphenols are complicated, and changes in surface tension alone cannot reliably predict the foaming properties of protein–polyphenol complexes. To investigate this further, the dilatational rheological behavior of the interfacial films formed by the WPI/GA complexes was analyzed.

According to the kinetic theory of interfacial adsorption, variations in the dilatational modulus (E) and the dilatational elasticity (E_d_) over time are related to the unfolding and rearrangement of active molecules at the interface. Changes in both the interfacial dilatational viscoelastic modulus and the elastic modulus with time are shown in Figure 4B,C. The values of E and E_d_ increased significantly over time, indicating that the adsorption of WPI/GA complexes at the interface increased, leading to a higher interfacial density. The E_d_ values were consistently much higher than the E_v_ values, suggesting that the interfacial films formed by the WPI/GA complexes predominantly exhibited elastic behavior. This result aligns with previous studies on the interaction between chicoric acid and β-lactoglobulin [26].

The final E and E_d_ values were higher than those of the other samples when the ratio of WPI/GA was 1:1, which indicated that the interfacial film formed by the WPI/GA complex had a strong elastic structure and thus had good resistance to external deformation. It has been previously reported that an interface with an elastic structure results in a system with a more stable foam structure [27], which is also consistent with previous foam stability results. To elucidate the role of the interaction between WPI and GA in the viscoelastic properties of the interfacial films further, the relationship between the dilatational viscoelastic modulus (E) and the surface pressure (π) was evaluated, and the results are shown in Figure 4D. In all of the samples tested, E increases with an increasing π, indicating that the interaction of the WPI or the WPI/GA complexes adsorbed at the interface is enhanced with time. This result is consistent with the earlier theory proposed by previous studies [28,29]. In the ideal interfacial adsorption curve for a single protein, the slope was kept as 1, while the slopes of the E-π curves of the tested samples in this study were all much larger than 1, which suggests that the interfacial adsorption behavior of the WPI/GA complex deviates from the ideal state to some extent and, on the other hand, implies that the interactions between the WPI/GA molecules at the interface were much stronger. Furthermore, the E-value of the WPI/GA complex was higher than that of the single WPI at the same surface pressure (π), which could be attributed to the more significant unfolding and subsequent rearrangement of the WPI molecules at the interface due to GA, in agreement with our previous conclusions on the interaction between GA and sodium caseinate [18]. Furthermore, the higher number of aromatic rings and hydroxyl groups in EGCG compared to that in GA promotes additional cross-linking via interactions between benzene rings and hydrophobic amino acids. This subsequently reduces the conformational rigidity of WPI at the interface, leading to a more pronounced loss of elasticity in the EGCG-treated samples relative to that in those treated with GA [27,30].

## 4. Foamability and Foam Stability

**Figure 5:**
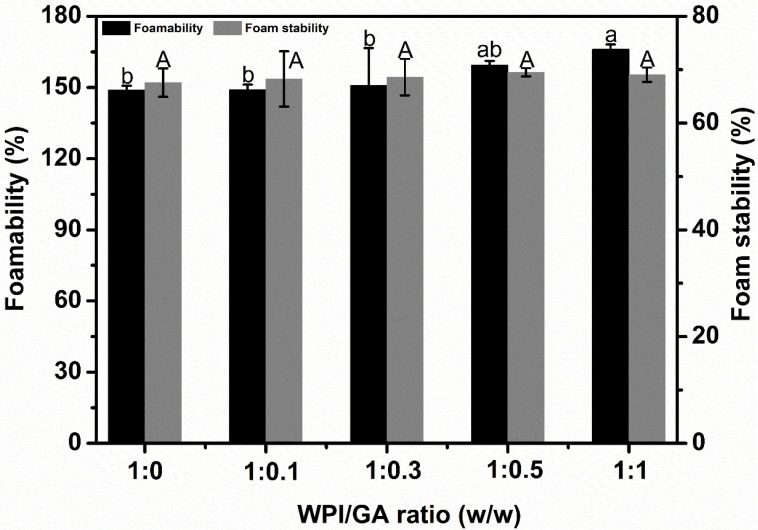
Foamability and foam stability as a function of different mass ratios for WPI/GA complexes. Means with different letters (A, a−b) differ significantly (P <0.05).

Figure 5 illustrates the differences in the foam properties of WPI/GA complexes with various mass ratios (1:0, 1:0.1, 1:0.3, 1:0.5, 1:1). The foamability of the WPI/GA complexes gradually increased with an increasing GA concentration. However, the foamability was considered to follow an upward trend when the WPI-to-GA ratio exceeded 1:0.5, according to the statistical analysis. Although this result did not directly correspond to the changes in interfacial tension, particle size played a significant role in the foamability of the complexes. Smaller particle sizes facilitate the rapid adsorption of the complex molecules at the interface, which contributes to the quicker formation of bubble structures.

As the GA concentration increased, the foam stability of the WPI/GA complex also gradually improved, although there were no statistically significant differences between the samples. Interestingly, the results on the interfacial dilatational modulus revealed that the interfacial film formed by the WPI/GA complex had the highest modulus at a WPI/GA ratio of 1:1. However, despite this finding, no significant difference in foam stability was observed in the macrofoam system. This discrepancy may be attributed to the change in the interfacial modulus not fully reflecting the macrofoam properties. Nonetheless, this phenomenon can be linked to the particle size results. The enhanced foam stability may be associated with the smaller diameter of the WPI/GA complexes, as smaller particles are more likely to be irreversibly adsorbed at the interface, leading to improved foam stability. Similar observations have been reported in other studies [14,25].

## 5. Conclusions

In this study, the interaction between WPI and gallic acid (GA) was investigated using spectroscopic techniques. The primary forces driving the interaction between WPI and GA include hydrogen bonding and hydrophobic interactions. In the bulk phase, GA binds to WPI molecules, forming a stable spacing that results in a smaller particle size for the WPI/GA complex compared to that of single WPI molecules. The binding of GA to WPI induces the unfolding of WPI molecules and exposes more hydrophobic groups on the surface, thereby increasing the hydrophobicity of the WPI/GA complex.

GA also influences the interfacial and foam properties of WPI. The ability of the WPI/GA complex to reduce surface tension increases with an increasing GA concentration, particularly at a WPI-to-GA ratio of 1:0.3, where the surface pressure (π) of the complex is at its lowest. In parallel, the foamability of the WPI/GA complex improves as the GA concentration rises. The binding of GA to WPI leads to the formation of stable viscoelastic films after the WPI/GA molecules adsorb to the interface, with the highest interfacial modulus (E-value) observed at a WPI/GA ratio of 1:0.3.

However, the foam stability of the complexes showed only a slight increase with higher GA concentrations, with no significant differences between the groups. This suggests that the foamability of the complex is primarily influenced by particle size, while foam stability is more closely related to the viscoelastic properties of the interfacial film and the interactions between WPI and GA.

Overall, this study demonstrates that the structural and interfacial properties of WPI can be modulated through the addition of polyphenols, such as GA, which could be useful for a range of applications involving interfaces, including foams and emulsions.

## Figures and Tables

**Table 1:** Fluorescence quenching parameters of WPI/GA complexes.

Mass Ratio	F_0_/F_280 nm_	*K_q_*/[L/(mol·s)]_280 nm_	F_0_/F_290 nm_	*K_q_*/[L/(mol·s)]_290 nm_
**1:0**	1		1	
**1:0.1**	1.1764	3.00 × 10^10^	1.0304	5.19 × 10^10^
**1:0.3**	1.6915	3.92 × 10^10^	1.2081	1.18 × 10^11^
**1:0.5**	2.3086	4.45 × 10^10^	1.4403	1.50 × 10^11^
**1:1**	4.4575	5.88 × 10^10^	1.9815	1.67 × 10^11^

## Data Availability

The original contributions presented in the study are included in the article; further inquiries can be directed to the corresponding authors.
